# Comprehensive Experimental, Theoretical, and Surface Characterization Studies on the Corrosion Inhibition Mechanism, Electrochemical Durability, and Long-Term Protection Ability of a 5‑Mercapto-1-Methyltetrazole Film on Mild Steel in 1 M HCl Solution

**DOI:** 10.1021/acsomega.6c06151

**Published:** 2026-08-25

**Authors:** Aleattin Çelik, Ramazan Solmaz

**Affiliations:** † 162312 Bingöl University, Science and Letters Faculty, Chemistry Department, Bingöl 12000, Türkiye; ‡ Bingöl University, Health Sciences Faculty, Occupational Health and Safety Department, Bingöl 12000, Türkiye

## Abstract

In this study, the adsorption behavior and corrosion inhibition performance of 5-mercapto-1-methyltetrazole (MMT) on mild steel (MS) were comprehensively evaluated in 1 M HCl solution by electrochemical, surface-characterization and quantum-chemical methods. In order to clearly demonstrate its novelty and suitability for practical applications compared to conventional short-term assessments, the electrochemical durability and long-term protective ability of the inhibitor film were examined. It was shown that the inhibition efficiency depends on MMT concentration and remains exceptionally high, reaching approximately 97.3% (LPR) and 95.8% (PDP) even after 120 h of continuous immersion. MMT acts as a mixed-type corrosion inhibitor with a predominant cathodic inhibition effect at low concentrations. The corrosion reaction is charge-transfer-controlled, and the MMT molecules form a barrier by adsorbing at the metal/solution interface. Thermodynamic calculations yield an adsorption free energy (Δ*G*_ads_) of −33.2 kJ/mol, which suggests that the adsorption process involves mixed physisorption and chemisorption contributions, with a tendency toward stronger chemical interactions. SEM and EDX mapping analyses confirm a uniform distribution of the MMT film across the steel surface. Rather than degrading over time, the MMT film dynamically rearranges and compacts. Potential of zero charge (PZC) measurements indicate that the metal surface carries an excess positive surface charge. The assembled surface film is electrochemically durable under both chronoamperometric and cyclic voltammetric conditions.

## Graphical abstract

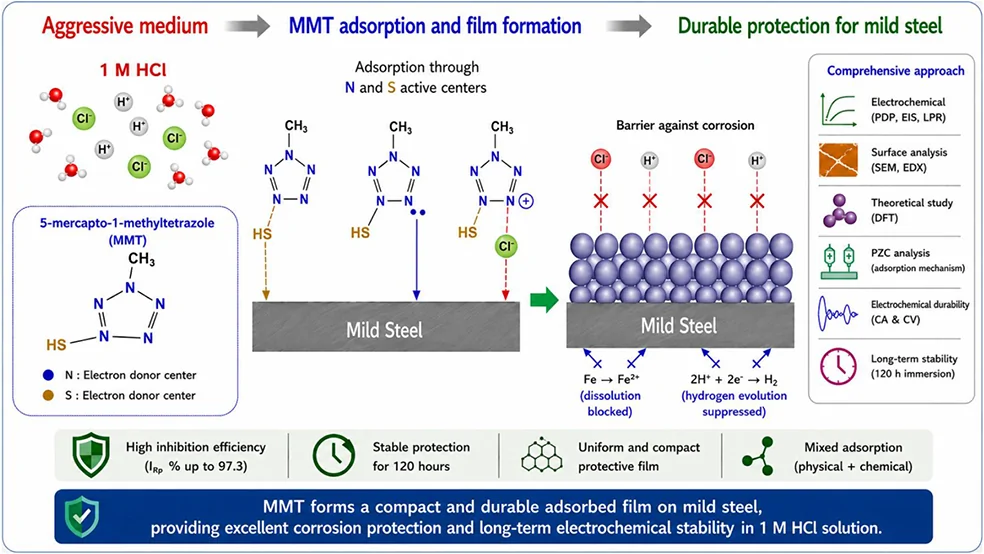

## 1 Introduction

Corrosion, widely considered one of the most destructive phenomena in the industrial field, causes serious structural and operational problems in many technological sectors.^1–3^ Worldwide economic losses due to corrosion are estimated to be between approximately 2% and 5% of the gross domestic product (GDP) of industrialized countries.^4–7^ Due to its low cost, abundant availability, ease of processing, and good mechanical properties, MS is the most widely used alloy in various industrial sectors including petroleum,^8^ chemical,^9^ automotive,^9,10^ and construction.^11^ However, a critical disadvantage of MS is its weak corrosion resistance, especially when exposed to highly aggressive aqueous environments.^12–15^ In industrial operations, strong mineral acids, most notably HCl and H_2_SO_4_, are extensively used for many purposes such as acid cleaning, descaling, and acidizing oil wells.^16–18^ These acidic conditions cause rapid corrosion of MS, leading to unpredictable safety risks and enormous economic losses worldwide, amounting to billions of dollars every year.^4,11,19–21^

Although various methods are available to reduce the corrosion rate, the use of corrosion inhibitors stands out as one of the most practical and cost-effective solutions due to their low concentration requirements and high efficiency.^22–25^ Historically, traditional inorganic salts have been used as corrosion inhibitors effectively in industry for many years.^26–28^ However, strict global ecological regulations have largely restricted their application because most of these heavy-metal compounds are highly toxic, carcinogenic, and ecologically devastating.^29–31^ Additionally, although inorganic inhibitors primarily act by forming a protective passive film on the metal surface and affecting the anodic reaction,^32–34^ these passive layers can be highly brittle and provoke dangerous localized attacks, such as pitting corrosion. Furthermore, their residues are difficult to eliminate, posing serious health and environmental risks.^34^ Consequently, modern corrosion science has decisively shifted toward the use of organic corrosion inhibitors to overcome these drawbacks.^35,36^ In particular, organic corrosion inhibitors exhibit high effectiveness at very low concentrations without requiring oxygen for passivation, slowing down or preventing the dissolution rate of metals in corrosive environments.^10,37,38^ The fundamental mechanism of action of organic corrosion inhibitors is based on spontaneous adsorption onto the metal surface. By establishing a densely packed protective film layer, they successfully interrupt contact between the metal and the aggressive environment.^39–41^ Ultimately, these molecules suppress the corrosion reaction by blocking the electrochemically active surface areas, drastically slowing down the dissolution rate.^24,37,42,43^ In recent years, the literature concerning MS corrosion in acidic environments has been significantly expanded; various classes of eco-friendly and synthetic organic compounds have been extensively investigated and proven to be highly effective in mitigating acidic corrosion.^44–46^

Fundamentally, the interfacial adsorption process begins with the replacement of preadsorbed water molecules on the metal surface by inhibitor molecules.^1,43,47^ For this process to occur spontaneously, it is strictly necessary that the inhibitor–metal interaction energy surpass the metal–water interaction energy.^47^ Once this energetic barrier is overcome, the adsorption of organic inhibitors on solid surfaces proceeds as a highly spontaneous process. This phenomenon is quantitatively evidenced by a negative standard free energy change (Δ*G*_ads_), which occurs via physical, chemical, or mixed-type mechanisms.^47–49^ Structurally, the superior ability of organic inhibitors in inhibiting corrosion is fundamentally based on the interaction of lone electron pairs from electronegative heteroatoms (N, S, O, P) in their molecular structures with empty orbitals on the metal surface, thereby forming an adsorption film.^23,41,50–52^

It was previously reported that 5-mercapto-1-methyltetrazole (MMT) protects A9M steel in a 0.1 N HCl medium with an inhibition efficiency of 86.87%.^53^ While certain tetrazole and mercapto derivatives have been reported for their corrosion protection properties on various metals, e.g., A9M steel,^53^ Q235 steel,^54^ mild steel,^55^ carbon steel,^56^ copper, and brass^57,58^ or as paint additives,^59^ existing literature predominantly focuses on short-term exposure conditions or relatively mild environments. However, the long-term stability and electrochemical durability of such inhibitor films are crucial to their industrial viability. Contrary to the literature, this study examined the corrosion of MS in a 1 M HCl solution using a variety of comprehensive techniques following both short-term and long-term exposure. A critical gap remains regarding these inhibitors’ true industrial applicability, particularly under conditions where MS is routinely subjected to highly aggressive 1 M HCl descaling solutions for extended periods. Although several azole derivatives exhibit satisfactory initial inhibition performance, the protective films of organic compounds are often susceptible to degradation or desorption during prolonged exposure to aggressive chloride-containing environments. This is one of the major issues with current inhibitor design, which can be successfully addressed by the unique molecular architecture of MMT. This compound contains several electron-donating nitrogen atoms in the tetrazole ring, together with an exocyclic sulfur atom that drastically enhances its adsorption capability, enabling strong coordination interactions between MMT and the vacant 3d orbitals of the iron surface.^54,60^ Furthermore, the presence of the hydrophobic methyl group helps in shielding the underlying metal from excessive water molecules in the bulk solution. Therefore, this study not only explores the adsorption and corrosion inhibition properties of MMT molecules on the MS surface but also provides a comprehensive evaluation of the dynamic electrochemical stability and durability of the MMT film formed on the metal under chronoamperometric and cyclic voltammetric conditions, together with a promising long-term protective performance.

Going far beyond just protecting metals, the strategic selection of MMT bridges the critical gap between industrial efficiency and environmental sustainability. Tetrazoles are widely recognized for their pronounced biological activities, and in general, exhibit significant potential in medicinal chemistry.^61–63^ With the use of these potentially less environmentally harmful, the severe threat of ecological damage caused by conventional inhibitors is completely bypassed. From an economic perspective, MMT has a straightforward and highly efficient synthetic route, ensuring its commercial viability.

While various tetrazole derivatives and MMT have been previously reported as corrosion inhibitors in short-term static assessments, the dynamic electrochemical durability of these films over prolonged exposure periods and their structural resilience against severe anodic and cathodic potential fluctuations have not been systematically addressed in the literature. Furthermore, establishing the initial adsorption sequence via the potential of zero charge (PZC) measurements is critical for a complete mechanistic understanding but remains highly scarce for tetrazole-based inhibitors. Therefore, the novelty of the present work lies in bridging this specific gap by demonstrating the high long-term interfacial stability of the MMT monolayer under extreme conditions and explicitly elucidating the chloride-bridged adsorption mechanism through a rigorous combination of long-term electrochemical, surface, and quantum chemical analyses.

The study aims to comprehensively investigate the adsorption and inhibition effects of MMT on MS corrosion in the 1 M HCl solution. The stability of the inhibitor film on the steel surface was examined under chronoamperometric (CA) and cyclic voltammetric (CV) conditions. Furthermore, the long-term protection ability and interfacial durability of the surface film were examined over a 120 h exposure period. For this aim, a robust combination of electrochemical measurements, advanced surface characterization techniques (SEM, EDX, AFM), and quantum chemical computations was systematically carried out.

## 2 Materials and Methods

### 2.1 Preparation of Electrodes

The MS working electrodes (WEs) were prepared by cutting the rod-shaped specimens into cylindrical sections of approximately 5 cm in length. Its chemical composition (wt%) was C (1.720), O (0.115), Al (0.173), Si (0.133), P (0.052), Cr (0.057), Mn (0.704), Ni (0.115), Cu (0.144), Mo (0.204), Sn (0.126), and Fe (remainder). To guarantee electrical conductivity, a copper wire was securely attached to one end, and embedded in a polyester resin block so that only the surface to be measured remained exposed. The surface area of the prepared electrodes that came into contact with the solution was 0.785 cm^2^. Prior to the measurements, the electrode surface was polished using abrasive papers of varying grain sizes (ranging from 320 to 1200 grit) with the help of a Mechanical Polisher (Imro Propol-VTD). After polishing, the electrodes were rinsed sequentially with deionized water, ethanol, and again with deionized water. Rather than a simple rinse, they were subsequently kept in an ultrasonic bath for a sufficient period to entirely eliminate any residual particles remaining on the surface from the polishing process. Following their removal from the ultrasonic bath, the electrodes were thoroughly washed once more with ethanol and deionized water, quickly dried with a clean filter paper, and immediately transferred into the test solution to prevent undesired atmospheric oxidation.

The Pt sheets with dimensions of 10 mm × 10 mm × 0.1 mm, providing a 2 cm^2^ total surface area, served as the counter electrode. Prior to measurements, the Pt surface was chemically activated and cleaned by soaking it in a 1:1 (v/v) HNO_3_:H_2_O solution for approximately 5 minutes. A commercial silver–silver chloride electrode (Ag/AgCl) with a 3 M KCl filling solution served as the reference electrode for the electrochemical measurements.

### 2.2 Test Solutions

All corrosion tests were performed in 1 M HCl solutions without and with the addition of various concentrations of MMT (0.1 mM, 0.5 mM, 1 mM, 5 mM, 10 mM). To facilitate industrial relevance and practical dosing applications, it should be noted that the selected molar concentrations of MMT correspond to approximately 11.6, 58.1, 116.1, 580.7, and 1161.4 ppm, respectively. The MMT compound was purchased from Aldrich (>98.0% purity) and used directly without any further processing. The corrosive medium, 1 M HCl solution, was prepared by diluting analytical-grade concentrated HCl (37%) with deionized water. Rather than initiating electrochemical measurements immediately, the working electrode (WE) was first immersed in the test solutions for 1 h. This crucial stabilization period ensures the attainment of a steady-state open-circuit potential (OCP). During all subsequent measurements, a thermostatically controlled water bath (Nüve) maintained the temperature of the test solutions at 298 ± 1 K under naturally aerated conditions. An identical protocol was applied to prepare the steel substrates for subsequent surface characterization studies.

### 2.3 Electrochemical Measurements

All electrochemical measurements were carried out using a computer-controlled potentiostat-galvanostat (CHI 660D) equipped with a conventional three-electrode setup. Before starting measurements, the WE was immersed in the test solution for 1 h to ensure that a steady-state open-circuit condition was reached. To verify this interfacial equilibrium, the change in OCP was continuously monitored versus exposure time (*E*_ocp_–*t*). EIS measurements were carried out at the stabilized *E*_ocp_, applying a 5 mV peak-to-peak AC sinusoidal amplitude over the frequency range from 100 kHz to 10 mHz. LPR measurements were subsequently performed by scanning the electrode potential from −10 mV to +10 mV vs *E*_ocp_ at a scan rate of 1 mV s^–1^. The polarization resistance (*R*_p_) of the MS was calculated from the slope of the obtained current–potential curves. Finally, PDP curves were obtained potentiodynamically by polarizing the working electrode from the cathodic direction toward more positive potentials at a constant scan rate of 1 mV s ^–1^.

In order to rigorously evaluate the stability of the inhibitor film formed on the metal surface, after the MS electrode was immersed in the test solution for 1 h, +100 mV anodic and −100 mV cathodic overpotentials (vs *E*_ocp_) were applied for 3600 s using chronoamperometry (CA), and the passing current density was monitored over time. Complementing this approach, potentiodynamic tests (cyclic voltammograms) were also obtained between *E*_ocp_ and either +100 mV anodic or −100 mV cathodic overpotentials (vs *E*_ocp_) at a scan rate of 10 mV s^–1^ to assess film endurance under severe potential fluctuations. For this aim, 20 sweep segments (10 complete cycles) were applied.

Determining the electrostatic nature of the metal/electrolyte interface is fundamental; therefore, the excess surface charge of the MS in the corrosive medium with the addition of 10 mM MMT (optimized concentration) was estimated by the determination of the potential of zero charge (*E*_pzc_). To this aim, *R*_p_ values for the steel were obtained at different potentials using the EIS technique, and the data obtained were plotted versus the applied potential. The excess surface charge of the metal was determined from the data obtained.

The prolonged stability of the surface inhibitor film and the ultimate protection ability of the inhibitor were extensively assessed over the exposure time. For this aim, the WE was immersed in the optimally inhibited solution (10 mM MMT) for 120 h and its *E*_ocp_ was monitored as a function of immersion time. At the same intervals, the LPR technique was used to determine the *R*_p_ values of the MS steel in the absence and presence of the optimized MMT concentration. After 120 h, PDP curves were obtained. To evaluate the stability of the inhibitor film, its protection ability, and the actual change in performance, identical parallel tests were repeated in the uninhibited 1 M HCl solution.

#### 2.3.1 Surface Characterization

The surface morphology of the steel samples was examined after exposing the MS specimens to the 1 M HCl solution without and with the optimum concentration of 10 mM MMT for 1 h and 120 h. The surface morphologies of the samples were examined by scanning electron microscopy (SEM) (JEOL 6510). The presence and distribution of the inhibitor film over the steel surface were assessed by energy-dispersive X-ray spectroscopy (EDX) (JEOL 6510). Specifically, EDX mapping was utilized to meticulously trace the distribution of N and S heteroatoms, which originate from the MMT molecular structure, confirming the formation of a densely packed, homogeneous protective layer over the metallic substrate.

#### 2.3.2 Theoretical Calculations

Quantum chemical computations based on Density Functional Theory (DFT) were executed to examine the intrinsic electronic properties of the MMT inhibitor molecule. DFT calculations were performed with the help of a Gaussian 09 program package (USA), employing the B3LYP exchange–correlation functional in conjunction with the 6-311++G­(d,p) basis set. Rather than merely reporting basic structural traits, we specifically targeted critical global reactivity descriptors, including the highest occupied molecular orbital energy (*E*_HOMO_), the lowest unoccupied molecular orbital energy (*E*_LUMO_), the energy gap (Δ*E*), absolute electronegativity (χ), and the precise fraction of transferred electrons (Δ*N*). By evaluating these specific indices, we aimed to explicitly map the donor–acceptor dynamics between the highly electronegative nitrogen and sulfur centers of MMT and the vacant 3d orbitals of the iron substrate, ultimately validating our experimental data within a quantum mechanical framework.

## 3 Results and Discussion

### 3.1 The Variation of Open-Circuit Potential with Exposure Time

The variation of *E*_ocp_–*t* provides significant insight into the adsorption of organic molecules on metal surfaces, the initiation and progression of the corrosion reaction, as well as the anodic or cathodic mechanism effects.^60–62^ The steady-state conditions, which are essential for the repeatability of the measurements, can also be clearly observed in these plots. To assess these interfacial dynamics, the *E*_ocp_–*t* curves of MS in the 1 M HCl solution, both in the absence and presence of the optimized concentration of 10 mM MMT, were recorded for 3600 s, and the data obtained are comparatively shown in Figure 1.

**Figure 1:**
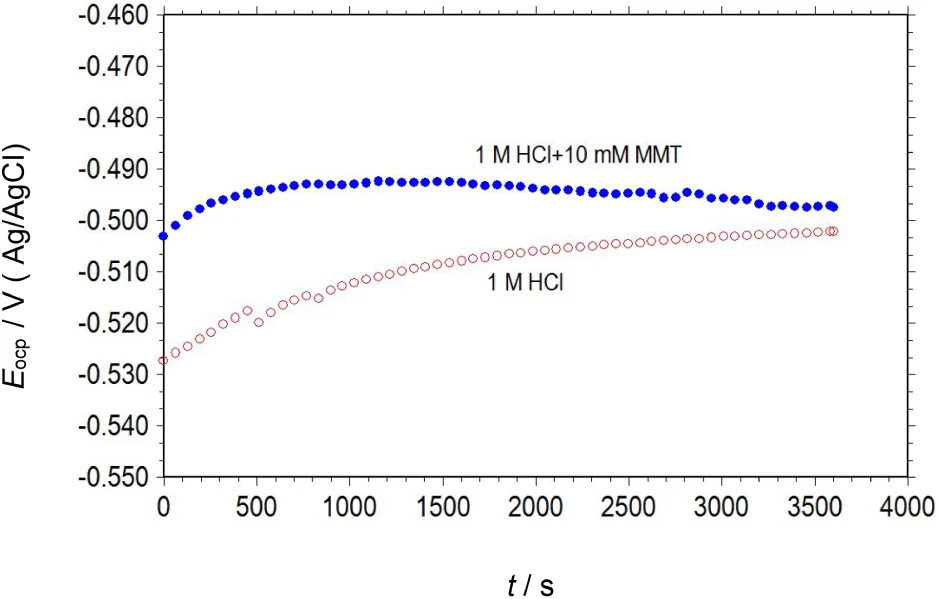
The variation of *E*_ocp_ of MS with exposure time in uninhibited 1 M HCl solution (○) and the inhibited solution containing the optimized concentration of 10 mM MMT (●).

As shown in Figure 1, in the absence of the inhibitor, the *E*_ocp_ of the MS electrode started at approximately −0.530 V and gradually shifted toward more positive (anodic) values over time. This shift was attributed to the dissolution of more active metal components on the steel surface, leaving behind more noble components^36,48^ or was associated with the rapid dissolution of the preexisting, air-formed oxide layer, which instantly exposed the highly active MS surface to aggressive chloride attacks. At the end of 3600 seconds, the potential remained nearly stable.

The addition of 10 mM inhibitor to the corrosive medium caused a further shift of the *E*_ocp_ toward more positive values. This outcome indicates the formation of an inhibitor film, which predominantly dominates the anodic reaction rather than the cathodic mechanism, and behaves as a protective barrier at the metal/solution interface.^63,64^ The initial *E*_ocp_ in the inhibited condition was approximately −0.504 V. Unlike the uninhibited system, the *E*_ocp_ reached a stable quasi-steady state remarkably faster, stabilizing almost entirely within the initial 500 s. This rapid stabilization of *E*_ocp_ clearly demonstrates that MMT molecules suggest rapid interfacial adsorption.^65^ After this immersion time, the fact that the *E*_ocp_ did not change considerably over time in the inhibitor-containing medium reflects the stability of the film formed on the metal surface. Exposing the metal to an aggressive HCl environment for an extended period to bring the OCP into equilibrium can, by their very nature, initially lead to surface damage and localized corrosion. However, when MMT is added to the corrosive environment, the potential reaches equilibrium rapidly equilibrium, demonstrating that the inhibitor immediately mitigates this initial damage and forms a protective film.

### 3.2 Potentiodynamic Polarization Studies

The potentiodynamic polarization (PDP) method is among the most reliable analytical methods for determining the kinetic mechanisms underlying cathodic hydrogen generation and anodic metal oxidation. Semi-logarithmic current–potential curves were obtained for an MS electrode in the 1 M HCl solution both without the inhibitor and with different concentrations of MMT after 1 h of immersion. The resulting curves are shown in Figure 2. From these plots, key electrochemical parameters including corrosion potential (*E*_corr_), corrosion current density (*i*_corr_), anodic Tafel slope (*b*_a_), cathodic Tafel slope (*b*_c_), theoretical weight loss (*W*), and inhibition efficiency (*I*_PDP_%) were determined and are listed in Table 1. The *I*_PDP_ % was calculated from the corrosion current densities using the following relation:

1 $$I_{\mathrm{PDP}}\%=\left(\frac{i_{\mathrm{corr}}-i_{\mathrm{corr}}^{\prime }}{i_{\mathrm{corr}}}\right)\times 100$$

**Figure 2:**
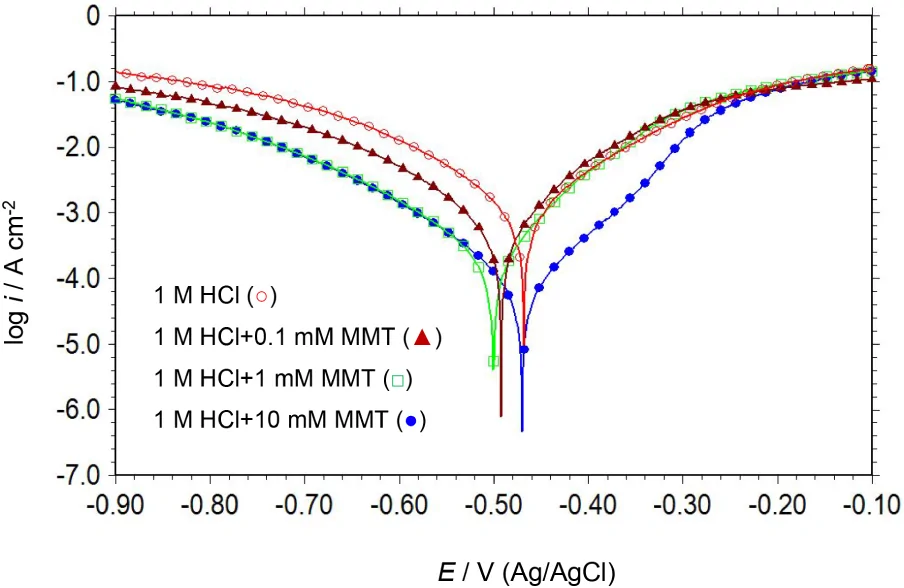
PDP curves of MS obtained in the test solutions after 1 h immersion.

**Table 1:** Electrochemical Parameters of the MS Electrode Derived from PDP Curves Recorded in the 1 M HCl Solution without and with the Addition of Various Concentrations of MMT at 298 K

*C* (mM)	*E*_corr_ (V)	*i*_corr_ (mA cm^–2^)	*b*_a_ (mV dec^–1^)	*b*_c_ (mV dec^–1^)	*W* (g m^–2^ h^–1^)	*I*_PDP_ %
1 M HCl	–0.468	1.1401	118	120	15.13	-----
0.1	–0.492	0.4289	100	114	5.69	62.4 ± 2.1
0.5	–0.501	0.2056	89	119	2.73	82.0 ± 1.8
1.0	–0.501	0.1492	88	117	1.98	86.9 ± 1.4
5.0	–0.495	0.1008	88	116	1.34	91.2 ± 0.5
10	–0.470	0.0595	77	116	0.79	94.8 ± 0.2

Here, *i*_corr_ and *i* ^′^_corr_ stand for the corrosion current densities of the MS electrode obtained in the 1 M HCl solution without and with the inhibitor, respectively. The *i*_corr_ values were derived by extrapolating the linear portions of the curves to the relevant corrosion potentials. The *W* values were calculated theoretically from the *i*_corr_ values using Faraday’s laws.

The PDP curve of the MS electrode reveals a typical Tafel behavior in 1 M HCl in both the anodic and cathodic regions.^60^ Under these aggressive conditions, the *E*_corr_ of MS is −0.468 V. The linearity observed in the Tafel regions of the *E*–log *i* curves indicates that the dissolution process is predominantly controlled by charge-transfer kinetics.^66,67^ In the absence of the inhibitor, progressive and uncontrolled metal dissolution leads to a continuous increase in the real effective surface area of the metal, which in turn accelerates the rise in the current density.^48^

A critical analysis of the PDP profiles and the derived kinetic data in Table 1 strongly indicates that the corrosion behavior of MS in the 1 M HCl solution is strongly influenced by the MMT concentration. Upon introducing MMT into the HCl solution, a simultaneous and drastic decrease in the current densities for both the anodic metal dissolution and the cathodic hydrogen evolution reactions is observed, as expected for an effective corrosion inhibitor. This situation indicates that MMT molecules effectively block the active sites on the metal surface. Compared with the uninhibited HCl solution, adding the inhibitor into the corrosive medium leads to a slight shift of *E*_corr_ toward more negative potentials at low concentrations. Therefore, MMT can be classified as a mixed-type inhibitor with a predominant effect on the cathodic reaction at lower concentrations.^64,66,67^

As the concentration of MMT increases, the inhibition efficiency improves progressively. This trend indicates that MMT molecules spontaneously adsorb onto the metal surface and increasingly block active sites on the metal.^60^ However, the impact of concentration is not identical for the anodic and cathodic processes. With increasing inhibitor concentration, the rate of the cathodic hydrogen evolution reaction decreases significantly, implying that a progressively larger fraction of cathodic sites becomes effectively covered by the adsorbed inhibitor molecules. On the other hand, increasing the concentration up to 5 mM does not lead to a substantial change in the anodic dissolution behavior. A marked decrease in the corrosion current density is observed when the concentration of MMT is increased to 10 mM. This kinetic response strongly suggests that a critical threshold concentration is required for sufficient coverage of anodic regions. Ultimately, the lowest corrosion current density and the highest inhibition efficiency were achieved at this optimum 10 mM MMT dosage. Considering both practical industrial applicability and economic viability, higher concentrations were not further investigated. At this optimized concentration, the *E*_corr_ of the steel did not change significantly with respect to that observed in the uninhibited HCl solution, which means that the inhibitor acts as a mixed-type corrosion inhibitor at this concentration.^14^ At high concentrations, the relatively large MMT molecules tightly and effectively cover the metal surface. This strongly anchored layer simultaneously blocks the active anodic sites to retard metal dissolution and restricts the diffusion and reduction of H^+^ ions at the cathodic sites. Therefore, the observed mixed-type behavior fundamentally arises from this simultaneous geometric blocking effect dictated by the comprehensive adsorption mechanism, rather than being governed by a strict potential shift.

In the *E*_ocp_–*t* (Figure 1) plot, the *E*_ocp_ of MS at 3600 s was more positive in the inhibitor-containing medium than in the inhibitor-free medium (Figure 1). However, the *E*_corr_ value observed from the PDP measurements is more negative compared to the inhibitor-free medium. Although this may appear to be a contradiction, it is a natural result of differences between the two methods. In the *E*_ocp_–*t* technique, *E*_ocp_ values are read under open-circuit conditions. However, in PDP measurements, the potential was obtained by scanning from more negative overpotentials to more anodic potentials. Consequently, in the cathodic region, before reaching the *E*_corr_ value, excess hydrogen gas formed on the electrode surface likely affects the surface film, displacing part of it from the surface. Additionally, in the DC technique, the application of potential affects the metal/solution interface. An identical observation has been reported in the literature.^14^

As can be seen from Figure 2 and Table 1, the cathodic current–potential curves are nearly parallel, and the cathodic Tafel slopes remain essentially unchanged across all MMT concentrations. This behavior indicates that the addition of MMT to the HCl solution does not alter the mechanism of the hydrogen evolution reaction.^20,61,63,68^ The inhibitor merely blocks the active sites through a simple blocking effect.^49,69^ Consequently, the reduction of H^+^ ions at the cathodic sites proceeds under charge-transfer control. In contrast to the cathodic response, the anodic Tafel slopes show a slight decrease with increasing inhibitor concentration. This concentration-dependent alteration implies that the inhibitor does not just physically block the sites, it actively modifies the anodic reaction pathway, thereby mitigating the overall corrosion process.^60^ As noted earlier, the suppression of the anodic metal dissolution reaction heavily depends on the concentration of the inhibitor.

As the applied potential is swept anodically from the corrosion potential, the current density increases; however, the magnitude of this increase remains noticeably lower in the presence of the inhibitor, particularly at the optimum 10 mM dosage. The inhibitor film acts as a protective barrier on the metal surface, and dissolution is largely confined to uncovered regions or to areas beneath the film. Interestingly, at this elevated inhibitor concentration, a pseudo-passive plateau appears within the potential range of approximately −0.420 V to −0.340 V. This feature is classically attributed to the accumulation of insoluble corrosion products beneath the organic framework, which temporarily plug the interfacial pores and synergistically enhance its protective character.^70^ Upon polarizing the electrode beyond this plateau, the current density rises sharply. At this stage, iron undergoes accelerated dissolution, leading to deformation and almost complete detachment of the protective film from the surface. This breakdown potential is commonly referred to as the desorption potential.^71^

The decrease in the corrosion rate with increasing MMT concentration can be assigned to the spontaneous adsorption of these heterocyclic molecules onto the MS surface and the formation of a protective inhibitor layer. The details of the adsorption process will be discussed comprehensively in the forthcoming sections.

### 3.3 Electrochemical Impedance Spectroscopy Studies

The Nyquist and Bode (log *f*–log *Z* and log *f*–*Phase*) plots obtained for the MS electrode in the 1 M HCl solution both without and with the inhibitor are presented in Figure 3a, b and c, respectively.

**Figure 3:**
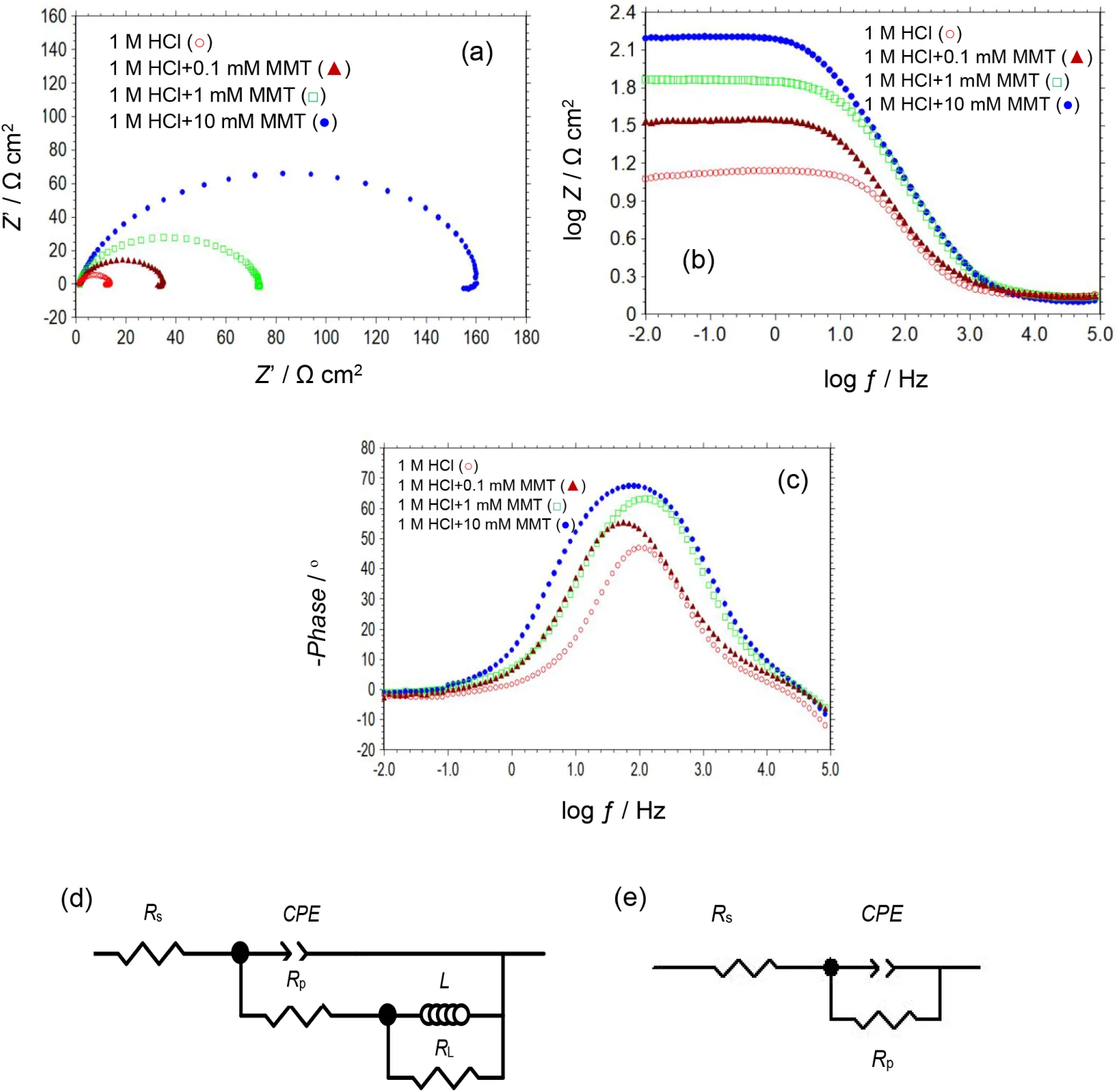
Nyquist (a) and Bode (log *f*–log *Z* (b) and log *f*–*Phase* (c)) plots of MS obtained in the test solutions; electrical equivalent circuit diagrams proposed for modeling the metal/solution interface; (d) for 1 M HCl, 0.1 mM, and 10 mM; (e) for 0.5 mM, 1.0 mM, and 5.0 mM.

In the absence of the inhibitor, a depressed capacitive semicircle is seen in the corresponding Nyquist plot in the high-to-medium frequency regions, immediately followed by an inductive loop at low frequencies. The low-frequency inductive loop in the absence of the inhibitor is generally related to the relaxation of adsorbed intermediate species (such as Cl_ads_^–^ and H_ads_^+^) and the unstable corrosion products (e.g., FeCl_2_). The observed behavior was supported by the corresponding Bode (log *f*–log *Z* and log *f*–*Phase*) plots, which indicate that the reaction is generally charge-transfer controlled, especially in the high- and intermediate-frequency regions. This complex electrochemical behavior was rigorously modeled using the electrical equivalent circuit diagram (EECD) presented in Figure 3d, and the corresponding impedance parameters are summarized in Table 2. Herein, a constant phase element (*CPE*), which is composed of *Y*_0_ (the proportionality factor) and *n* (the phase shift, which is a degree of deviation from ideality and is generally associated with the heterogeneity or roughness of the metal surface), was used instead of an ideal double-layer capacitance (*C*_dl_) to model the MS/HCl solution interface more accurately.^48^ The primary depressed semicircle observed at higher frequencies is fundamentally associated with the charge transfer resistance (*R*_ct_) and the resistance of the diffuse layer (*R*_d_). This distinct capacitive behavior is a classical indicator of an activation-controlled process at the metal/solution interface.^48^ The fact that the observed loop deviates from pure capacitive ideality, which is theoretically expected, can be associated with the inhomogeneity of the metal surface and the resulting frequency dispersion.^48,64^ On the other hand, the inductive loop appearing in the low-frequency region suggests that the species formed at the metal surface are thermodynamically unstable and tend to desorb or rapidly diffuse away from the interface over time. Furthermore, the accumulation of inhibitor molecules and insoluble corrosion products (*R*_a_) also synergistically contributes to the total barrier effect. These observations are comparable with the literature.^36^ Herein, *L* refers to the interfacial inductance. Because the resistance of the low-frequency inductive loop is exceptionally low, it is mathematically negligible. Therefore, in this study, the total resistance was defined as polarization resistance, *R*_p_ (*R*_p_ = *R*_ct_ + *R*_d_ + *R*_a_).^14,36^

**Table 2:** EIS Data for MS Obtained in the 1 M HCl Solution without and with Various Concentrations of MMT at 298 K after 1 h Exposure

*C*_inh_ (mM)	*CPE, Y*_o_ (10^–6^/ Ω^–1^ s^n^ cm^–2^)	*n*	*R*_p_ (Ω cm^2^)	*L* (H)	*R*_L_ (Ω cm^2^)	α	χ^2^	*I*_Rp_%
1 M HCl	758	0.89	10.8	4.13	1.59	–0.63	0.011	-----
0.1	1058.6	0.82	32.9	0.57	4.39	–0.78	0.009	67.2 ± 1.7
0.5	427.7	0.86	55.9	-----	-----	–0.77	0.005	80.7 ± 1.3
1.0	352.9	0.87	71.4	-----	-----	–0.76	0.002	84.9 ± 1.3
5.0	218.5	0.89	130.2	-----	-----	–0.72	0.010	91.7 ± 0.5
10	339.8	0.86	154.2	27.8	9.21	–0.68	0.040	93.0 ± 0.2

As can be seen from Figure 3, the addition of the inhibitor to the HCl medium does not significantly alter the overall capacitive shape of the impedance spectra; however, their radii change considerably depending on the MMT concentration. With systematically increasing MMT concentration, the size of the semicircles increases accordingly. Since the high- and middle-frequency loops are proportional to *R*_p_, this observation indicates that MMT molecules adsorb onto the metal surface. By forming a densely packed protective film, the inhibitor drastically reduces the number of active sites exposed to the acidic medium, thereby effectively lowering the corrosion rate. Interestingly, the main appearance of the Nyquist plots was highly dependent on the MMT concentration. At the lowest (0.1 mM) and highest (10 mM) boundaries, a small inductive loop appeared at low frequencies, resembling the behavior of the uninhibited system. At the lowest concentration (0.1 mM), the inhibitor coverage is insufficient to form a completely compact and isolated barrier. Consequently, the inductive loop at 0.1 mM MMT concentration arises from the same phenomena observed in the uninhibited solution. However, the reappearance of the inductive loop at the highest concentration (10 mM MMT) originates from distinctly different interfacial dynamics. This inductive behavior is fundamentally attributed to the relaxation process of densely adsorbed bulky MMT molecules and the dynamic adsorption/desorption equilibrium of the metal–organic complexes (Fe-MMT) formed at the highly packed metal/solution interface. This specific dynamic state system was precisely modeled by the EECD presented in Figure 3d. Conversely, only a single capacitive time constant was observed for the intermediate concentrations, specifically 0.5 mM, 1.0 mM, and 5.0 mM. This metal/corrosive-medium system was modeled by the EECD given in Figure 3e. The corresponding Bode plots support these observations. The related log *f*–log *Z* plot (Figure 3b) shows that there is a marked enhancement of the low-frequency impedance (log *Z*) values with respect to MMT concentration, indicating increased corrosion resistance. In line with this, as seen from the log *f*–*Phase* plot (Figure 3c), the phase-angle peak becomes wider and moves toward negative values at intermediate frequencies when MMT is added. This observation indicates a capacitive behavior and clearly verifies the successful formation of a dense, stable, and protective MMT barrier layer strongly adsorbed on the mild steel surface. The lower phase angle of 90° was generally ascribed to the nonideal capacitance and the presence of inhomogeneities in the system due to the inhibitor and/or corrosion products at the metal/solution interface.^72^

The inductive loop observed at low frequencies disappears at these optimum intermediate concentrations, e.g., at 1 mM MMT, while in other cases its contribution is mathematically negligible compared to the total resistance and has therefore been disregarded. By ignoring *R*_L_, the total resistance of the high- to intermediate-frequency loop is denoted as the *R*_p_, which is the sum of *R*_ct_, *R*_d_, and *R*_a_ and the film resistance (*R*_p_ = *R*_ct_ + *R*_d_ + *R*_a_ +*R*_f_).^48,60^ The EIS data were analyzed by fitting the experimental data to the proposed EECDs using a licensed ZView Software, and the data obtained are presented in Table 2.

The inhibition efficiency (*I*_Rp_ %) from the EIS measurements was calculated using the following relationship, where *R*_p_ and $R_{p}^{\prime }$ represent the polarization resistances in the absence and presence of the inhibitor, respectively.

2 $$I_{Rp}\%=\left(\frac{R_{p}^{\prime }-R_{p}}{R_{p}^{\prime }}\right)\times 100$$

The data obtained from EIS are consistent with the results of the PDP measurements. The *n* value remains close to 0.90 on average suggesting that the MMT molecules successfully displace corrosive species to form a relatively compact and well-organized molecular film on the surface, effectively sealing the interfacial defects and balancing the surface roughness.^48^ The slopes of the middle-frequency regions of log *Z*–log *f* plots (linear part) (α) ranges between −0.63 and −0.78, which is due to a nonideal metal/solution interface.^36^ At the optimum MMT concentration of 10 mM, the *CPE* (*Y*_0_) value decreased by more than 50% compared to the uninhibited corrosive medium. This reduction can be attributed to the adsorption of inhibitor molecules onto the surface, leading to increased surface coverage and/or an increase in the thickness of the electrical double layer at the metal/solution interface.^73^ The adsorption of MMT molecules, which are larger than water, onto the metal surface occurs through the displacement of previously adsorbed water molecules.^74–76^ This process results in a decrease in capacitance and a corresponding increase in the electrical double-layer thickness. According to the Helmholtz model, the inverse relationship between the double layer capacitance and the adsorbed film thickness is expressed as follows:^77,78^

3 $$C=\frac{\varepsilon^{0}\varepsilon S}{d}$$

In this equation, *C* is the interfacial capacitance associated with the inhibitor-adsorbed metal/solution interface, *d* is the effective thickness of the inhibitor film within the electrical double-layer region, ε_0_ is the vacuum permittivity, ε is the dielectric constant of the interfacial medium, and *S* is the exposed surface area. Because the organic MMT molecules possess a significantly lower dielectric constant than the displaced water molecules, their continuous accumulation simultaneously thickens the barrier and reduces ε, resulting in the observed drastic drop in capacitance and superior surface coverage.^78,79^

### 3.4 Linear Polarization Resistance Studies

The *R*_p_ values for the MS electrode in the 1 M HCl solution with and without the inhibitor were determined from the inverse slopes of the corresponding current–potential curves using the equation given below:

4 $$R_{p}=\frac{\Delta E}{\Delta i}$$

The corresponding inhibition efficiencies (*I*_Rp_%) were calculated using eq 2. The results obtained from these data are summarized in Table 3. The data obtained show that the *R*_p_ and *I*_Rp_% values are in good agreement with the data obtained from the EIS measurements. The results indicate that the inhibitor molecules form a protective film on the metal surface, thereby increasing the corrosion resistance. Even when a small dose of MMT (0.1 mM) was added to the 1 M HCl solution, the electrode’s resistance to acidic attack increased instantaneously to approximately three times its original value. As the MMT concentration was systematically increased, *R*_p_ and *I*_Rp_% increased with increasing concentration, reaching maximum values of 159.9 Ω cm^2^ and 92.3%, respectively, at the optimum 10 mM concentration. The enhanced protection ability likely comes from the adsorption of MMT molecules onto the MS surface and the formation of a protective layer.

**Table 3:** Electrochemical Parameters of MS Derived from LPR Data Recorded in the 1 M HCl Solution in the Absence and Presence of Various Concentrations of MMT at 298 K

*C* (mM)	*R*_p_ (Ω cm^2^)	*I*_Rp_ %
1 M HCl	12.35	-----
0.1	34.6	64.3 ± 1.2
0.5	64.8	80.9 ± 0.8
1.0	78.6	84.3 ± 1.2
5.0	128.9	90.4 ± 0.5
10	159.9	92.3 ± 0.3

### 3.5 Surface Characterization Studies

To evaluate the morphological changes on the MS electrode surface, the specimens were immersed in the 1 M HCl solution both in the absence and presence of a 10 mM inhibitor for a duration of 1 h. The SEM images of the MS samples exposed to the 1 M HCl solution in the absence and presence of various MMT concentrations after 1 h immersion are shown in Figure 4.

**Figure 4:**
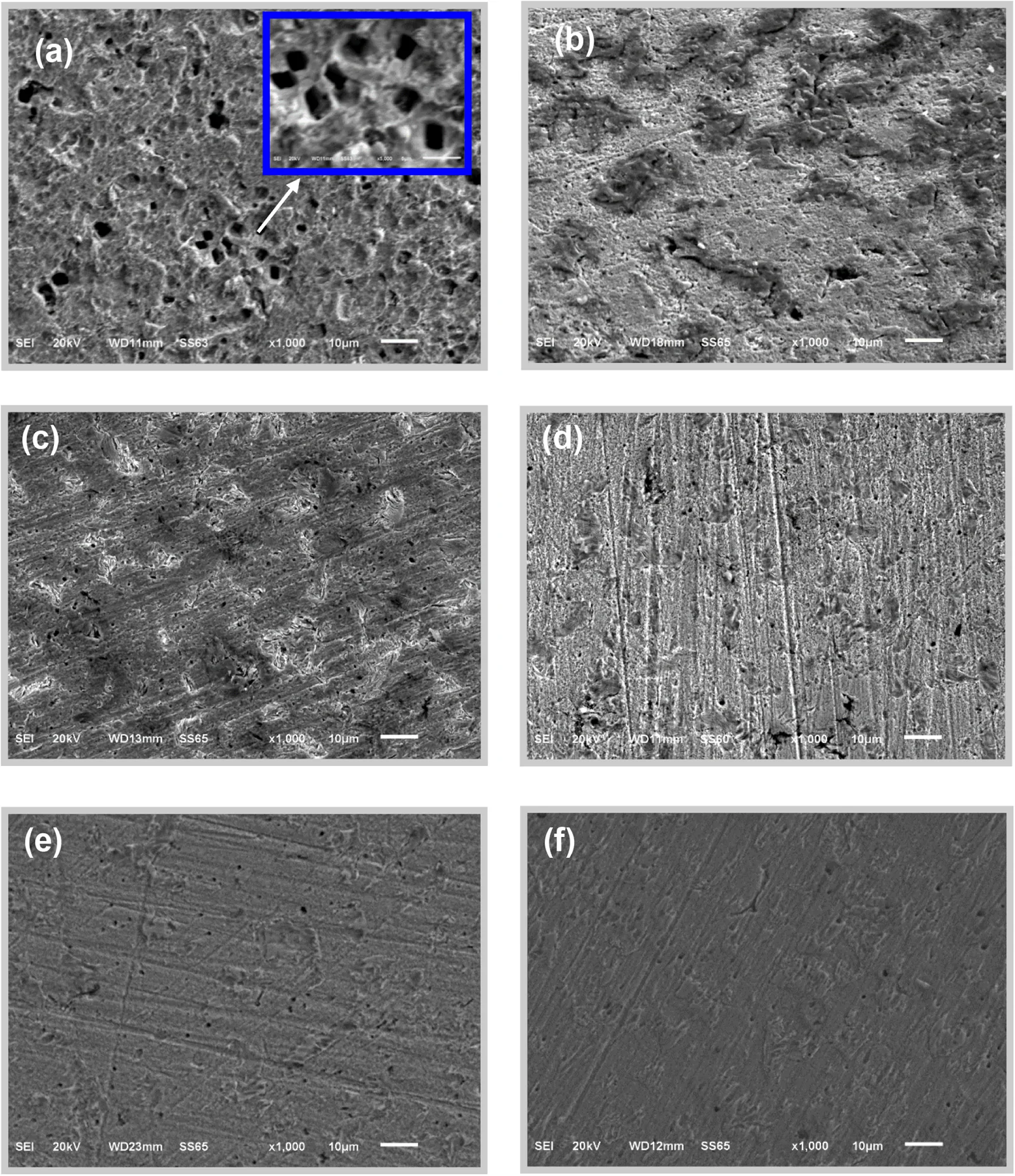
SEM images of the MS surface after exposure to the 1 M HCl solution in the absence (a) and presence of 0.1 mM (b), 0.5 mM (c), 1 mM (d), 5 mM (e), and 10 mM MMT (f) for 1 h.

As presented in Figure 4a, the metal surface in the inhibitor-free medium appeared to be severely and heterogeneously corroded. The presence of wide pits with varying diameters and depths indicates that the MS surface underwent intense, chloride-induced localized pitting corrosion.^48^ Conversely, after adding MMT to the corrosive environment, the metal surface is covered with a protective film, anodic dissolution is reduced, and the surface morphology becomes smooth and free of such localized chloride-induced defects. This protective film formation is strictly concentration-dependent; increasing the inhibitor concentration directly results in a structurally superior film. At the optimized 10 mM concentration (Figure 4f), the severity and density of the pits are substantially minimized, resulting in a significantly smoother surface compared to the blank sample (Figure 4a). However, it should be realistically acknowledged that since the inhibition efficiency is highly optimal but not absolutely 100%, minor localized damage and slight mass loss cannot be completely eliminated under such highly aggressive acidic conditions. Nevertheless, the morphological evidence confirms the outstanding protective nature of MMT.

To further validate the chemical identity of the protective film observed in the SEM micrographs, EDX analysis was performed. It is fundamentally important to acknowledge that EDX has inherent penetration depth limitations. Therefore, the EDX analysis herein is not intended to independently confirm the exact molecular structure of the subnanometer inhibitor film, but rather serves as a qualitative and complementary tool. The relative detection of heteroatoms (such as N and S) alongside the thermodynamic, PZC, and EIS findings collectively supports the presence of the MMT layer on the steel surface. Specifically, the spatial distribution of S and N atoms, key constituents of the inhibitor molecule, on the metal surface was evaluated using EDX mapping. It is important to note that the MS substrate inherently does not contain N in its elemental composition. Consequently, the detection of these specific heteroatoms serves as evidence of MMT adsorption. As it is seen in Figure 5, both S and N atoms, and thus the MMT molecules, are distributed uniformly across the metal surface with remarkable homogeneity and a dense film rather than being localized. Line-scan analysis of the same surface for the N and S elements further confirms this observation. Such a uniform distribution is a critical factor in ensuring comprehensive corrosion inhibition, as it confirms that the entire electroactive surface area is effectively protected against acidic degradation by the assembled molecular MMT film.

**Figure 5:**
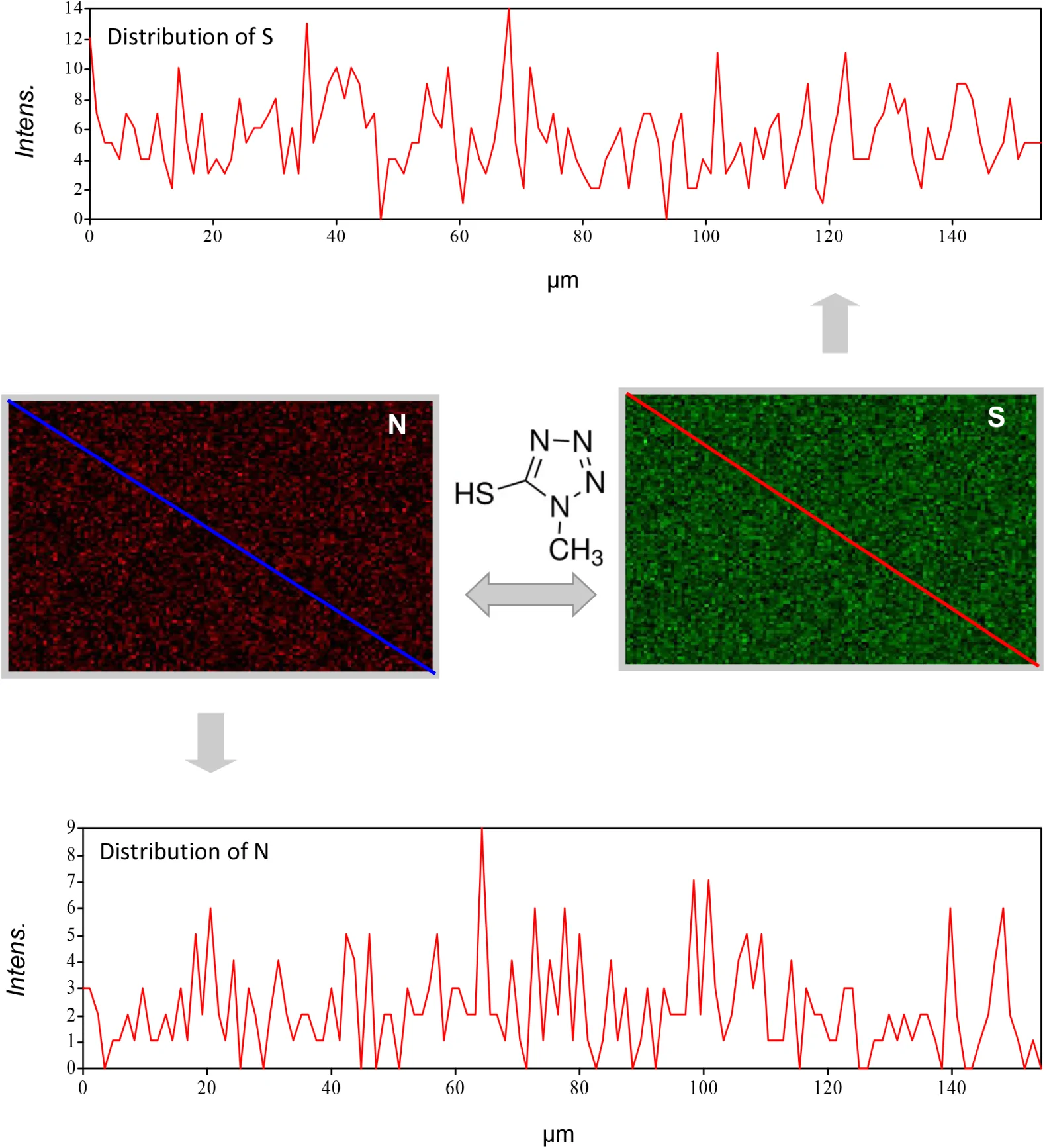
EDX mapping images of MS (the distribution of N and S elements on the surface) after 1 h of exposure to the 1 M HCl solution in the presence of 10 mM MMT; line-scan analysis of the same surface for N and S elements.

### 3.6 The Electrochemical Stability of the Inhibitor Film

Most conventional corrosion studies restrict their evaluations to static, steady-state exposures, completely ignoring the severe potential fluctuations typical in actual industrial descaling operations. To challenge the true electrochemical resilience of the self-assembled MMT film, the inhibited system was subjected to controlled anodic and cathodic potential perturbations using chronoamperometry (CA) and cyclic voltammetry (CV). In the CA measurements, constant anodic and cathodic overpotentials of ±100 mV (vs *E*_ocp_) were applied, and the resulting currents were continuously monitored as a function of time. The data obtained are presented in Figure 6. As can be observed from the figure, in the absence of the inhibitor, considerably higher current densities were recorded under both anodic and cathodic polarization conditions. In particular, the anodic current density exhibited a progressive increase over time, particularly during the initial 1000 s, which can be attributed to the rapid dissolution of the MS surface that continuously increases the real electroactive surface area as the metal degrades. In contrast, upon the addition of the inhibitor to the medium, both the anodic and cathodic current densities were significantly suppressed. Under both applied overpotentials, the current densities remained nearly constant over the entire 3600 s period. These findings indicate that the MMT film formed on the WE surface is highly durable and maintains its stability even over prolonged exposure, offering consistent protection against corrosive attack. We can fundamentally explain the film’s resistance to degradation by the molecular resistance exhibited by the bonds formed between the sulfur and tetrazole nitrogen (N) atoms of the MMT molecule and the vacant d-orbitals of iron, which successfully withstand severe anodic dissolution forces and cathodic hydrogen gas evolution.

**Figure 6:**
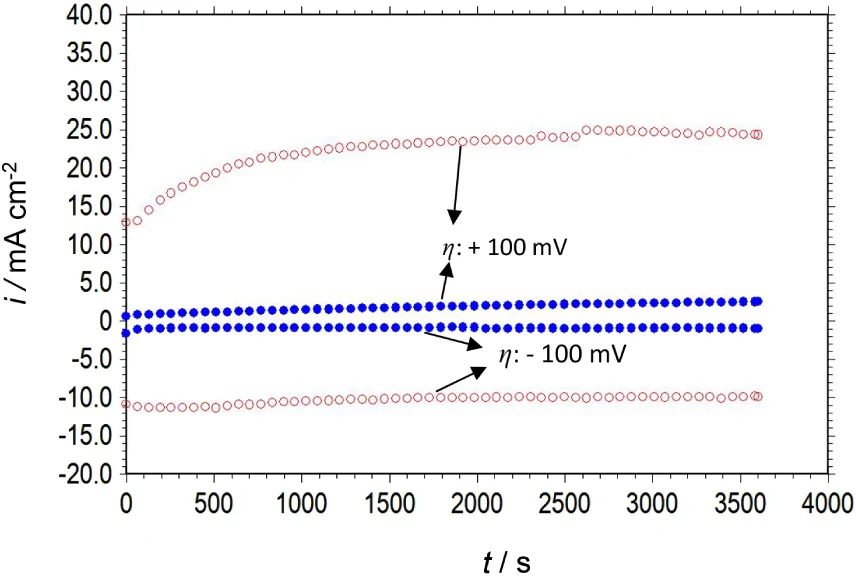
Chronoamperometric curves (*i–t*) of MS at +100 mV anodic and −100 mV cathodic overpotentials (vs *E*_ocp_) obtained in the 1 M HCl solution in the absence (○) and presence of 10 mM inhibitor (●).

To further validate this dynamic durability, cyclic voltammograms (CVs) were recorded by continuously sweeping the potential between *E*_ocp_ and +100 mV anodic or −100 mV cathodic overpotentials at a scan rate of 10 mV s^–1^. The results are presented graphically in Figure 7. As can be clearly seen from the obtained curves, the addition of the inhibitor leads to a substantial reduction in the current densities under both anodic and cathodic overpotential conditions. Even after 10 successive cycles (20 sweep segments), the organic film retains its durability with only negligible current changes, indicating an exceptional degree of interfacial stability. A slight increase in the current density observed during repetitive cycling is attributed to a modest rise in the dissolution rate of the iron beneath the molecular film. In contrast, during the cathodic sweeps, a modest reduction in the current density was observed in both the inhibited and uninhibited systems. This specific deceleration was ascribed to the vigorous generation of hydrogen gas and its insufficiently rapid diffusion away from the metal/solution interface, which created a localized physical hindrance, thereby limiting the recorded current density.^70^ This outstanding dynamic durability proves a critical mechanistic point: the MMT molecules do not merely rest on the surface. Rather, they construct an electrochemically robust, highly dense barrier that firmly withstands intense anodic oxidation forces and severe cathodic bubbling without evidence of major desorption or structural breakdown. Similar behavior has been reported for stable organic films under dynamic polarization conditions.^16,36,60^

**Figure 7:**
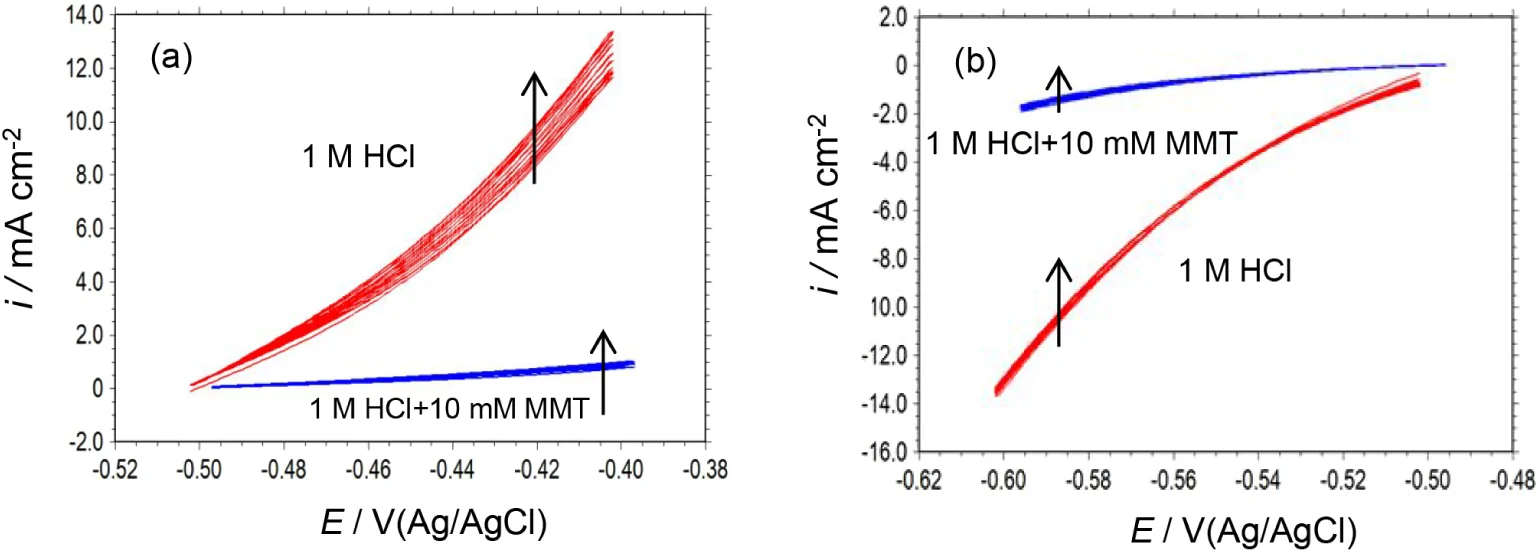
Anodic (a) and cathodic (b) cyclic voltammograms of MS at +100 mV anodic and −100 mV cathodic overpotentials (vs *E*_ocp_) obtained in the 1 M HCl solution in the absence and presence of 10 mM inhibitor (scan rate: 10 mV s^–1^, 20 segments (10 cycles)).

### 3.7 Long-Term Stability and Inhibition Performance over Extended Exposure

To evaluate the true industrial viability and time-dependent inhibition performance of MMT on the corrosion of the MS in the 1 M HCl solution, an extended evaluation was systematically conducted through *E*_ocp_–*t* monitoring, LPR, PDP measurements, and SEM analysis. For this purpose, the MS electrodes were immersed in the 1 M HCl solutions in the absence and presence of the optimized 10 mM inhibitor concentration, and *E*_ocp_ *–t* curves were continuously recorded at various immersion intervals. At the same intervals, the LPR technique was used to determine *R*_p_ of the MS in the absence and the presence of the inhibitor. *I*_Rp_% values were calculated using eq 2 and plotted versus exposure time (*t*–*I*_Rp_%). After 120 h of exposure, PDP measurements were conducted, and the resulting surface morphologies were examined by SEM. The data obtained are illustrated in Figure 8.

**Figure 8:**
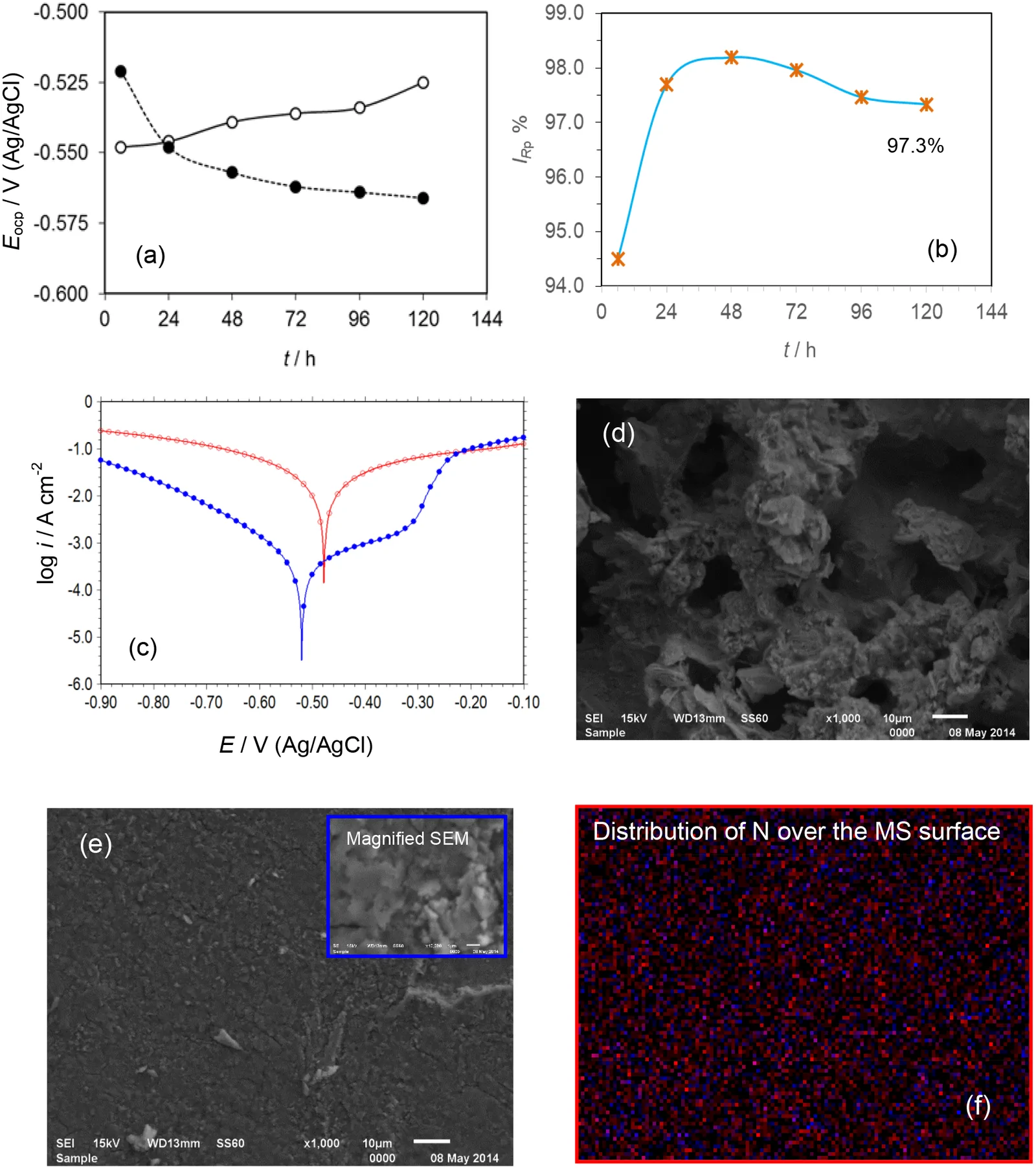
The variations of *E*_ocp_–*t* (a) and *t*–*I*_Rp_% (b) versus exposure time, and PDP (c) plots of MS obtained in the uninhibited 1 M HCl solution (○) and the inhibited solution with the addition of 10 mM MMT (●) after 120 h; SEM images of MS exposed to uninhibited (d) and inhibited (e) 1 M HCl solution for 120 h (a magnified SEM (10,000×) image was provided as an inset); the distribution of the N element over the MS surface, which was exposed to the 1 M HCl solution in the presence of 10 mM MMT for 120 h (f).

The variation of the *E*_ocp_ of the MS with immersion time in both the inhibitor-free and inhibitor-containing media is presented in Figure 8a. An inspection of the obtained plots reveals that, in the inhibitor-free medium, the *E*_ocp_ continuously moves to more anodic potentials over 120 h. This change in potential can be explained by the rapid dissolution of the metal in a corrosive environment over time and the accumulation of the resulting corrosion products at the metal/solution interface, where they act as a physical barrier. In the presence of the optimized 10 mM inhibitor concentration, the *E*_ocp_ of the MS electrode starts from more positive values and shifts progressively toward more cathodic regions, confirming domination of the inhibitor on the cathodic hydrogen evolution reaction during the long exposure time. Identically, the variation of the *I*_Rp_% versus exposure time was determined and is presented in Figure 8b. Apparently, the protection ability sharply increases during the initial time and reaches 94.5% and 97.7% after 6 h and 24 h, respectively. This observation indicates that the formation or thickening of the MMT film on MS increases progressively. The protection ability reaches its maximum value (98.2%) at 48 h. However, after 48 h of exposure, there is a slight reduction in the efficiency of inhibition up to 96 h. After this time, the efficiency almost remained constant at around 97.3%. Nevertheless, this inhibition efficiency remains high over the entire duration of testing. This observation can be attributed to possible partial desorption of some inhibitor molecules adsorbed on the surface, competitive adsorption by aggressive chloride ions, or degradation of the film due to prolonged exposure to the acidic solution. But, the efficiency is significantly high even after long exposure times, which clearly shows that the adsorbed MMT film does not lose its integrity and its effectiveness as a protective layer even after a long immersion time. This constant high efficiency indicates that the protective film provided by MMT is stable in the 1 M HCl solution.

The PDP curves obtained after 120 h are shown in Figure 8c. These curves exhibit a protective behavior comparable to that observed after shorter immersion times (e.g., 1 h in Figure 2). In the presence of the inhibitor, current densities are significantly reduced, while the corrosion potential shifts toward more negative values. These results suggest that after prolonged exposure, MMT behaves as a mixed-type inhibitor with a more pronounced effect on the cathodic reaction.^20,62^ This phenomenon is consistent with the trends observed in the *E*_ocp_–*t* studies, but opposite to that observed at the shorter exposure time (Figure 1). Using eq 1, *I*_PDP_% was calculated to be 95.8%. The slight difference in inhibition efficiencies observed after 120 h using the PDP and LPR techniques arises from the characteristics of the two techniques. This transition indicates that over the 120 h exposure period, the adsorbed MMT molecules undergo a structural reorganization and interfacial thickening, establishing a formidable physical barrier that predominantly suppresses the cathodic hydrogen evolution reaction.

The SEM images of MS obtained after being exposed to the 1 M HCl solution in the absence and presence of 10 mM MMT for 120 h are presented in Figure 8d and e, respectively. When compared with the SEM images recorded after 1 h (Figure 4), a substantial increase in both the size and depth of the pits is evident in the uninhibited medium, indicating the severe, unchecked progression of localized corrosion. In contrast, in the presence of the inhibitor, the MMT film appears to become denser over time. The distribution of the N element on the surface was analyzed by EDX mapping analysis and is shown in Figure 8f. As can be clearly seen from the resulting EDX mapping, the heteroatom markers of the MMT molecules are distributed uniformly across the metal surface, supporting the formation of a homogeneous and exceptionally durable protective film against the aggressive electrolyte even after 120 h of continuous acid attack.

### 3.8 Adsorption Isotherm

Organic molecules generally exert their influence by adsorbing onto metal surfaces. Therefore, understanding the adsorption mechanisms involved is of great importance. To fundamentally comprehend the interfacial binding dynamics and the specific adsorption mechanism of the inhibitor, various adsorption isotherms were tested using the experimental EIS data. The best fit was obtained with the Langmuir isotherm model (Figure 9).

**Figure 9:**
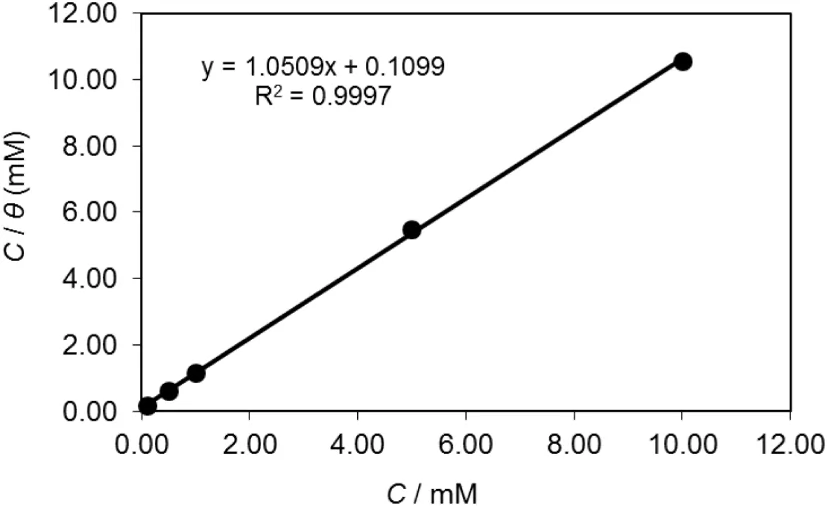
Langmuir adsorption plot of MS obtained in the 1 M HCl solution containing various concentrations of MMT.

The Langmuir isotherm is described by the following equation:

5 $$\frac{C_{(\mathrm{inh})}}{\theta }=\frac{1}{K_{(\mathrm{ads})}}+C_{(\mathrm{inh})}$$

where *C*_inh_ is the inhibitor concentration and *K*_ads_ is the adsorption–desorption equilibrium constant. The relationship between *K*_ads_ and the standard free energy of adsorption (Δ*G*°_ads_) is given by the following equation.

6 $${{\Delta G}^{{}^{\circ}}}_{\mathrm{ads}}=-RT\ln\left(55{.5K}_{\mathrm{ads}}\right)$$

Here, 55.5 represents the molar concentration of water in the solution (mol L^–1^), *R* is the universal gas constant, and *T* is the absolute temperature. The slope (∼1.07) and the linear regression coefficient (*R* ^2^ = ∼0.9999) values are remarkably close to unity, confirming that the adsorption of MMT molecules from the HCl solution onto the mild steel surface follows the Langmuir isotherm. Slight deviations from unity may indicate surface heterogeneity and possible interactions between adsorbed species. Accordingly, MMT molecules are assumed to form a highly organized monolayer inhibitor film on the metal surface.

From the adsorption isotherm, the *K*_ads_ value was calculated to be 12121 M^–1^. The positive and large *K*_ads_ values indicates strong adsorption of MMT on the metal surface. Using the relevant thermodynamic equation, the Δ*G*°_ads_ value was found to be −33.2 kJ mol^–1^. It is well known that the spontaneous adsorption of organic molecules onto metal surfaces is indicated by the negative Δ*G*°_ads_. Δ*G*°_ads_ values between −20 kJ mol^–1^ and −40 kJ mol^–1^ suggest that the adsorption of MMT on the MS surface follows a comprehensive mixed-type adsorption mechanism.^14,62,80^ However, since the Δ*G*°_ads_ value obtained for MMT (−33.2 kJ mol^–1^) is closer to −40 kJ mol^–1^ than to −20 kJ mol^–1^, the physical and chemical interactions are not expected to contribute equally. Instead, the adsorption process is better described as involving both mechanisms, with a slight predominance of chemisorption. It is highly probable that the adsorption process initiates with rapid physical attraction. Subsequently, as the molecules approach the surface closely, a prominent chemisorption phase occurs through the donor–acceptor interactions, involving the sharing of lone electron pairs from the S and tetrazole N atoms with the vacant d-orbitals of iron. This coordinated binding displaces the previously adsorbed water molecules, forming a highly stable, tightly packed protective monolayer. In addition, electrostatic interactions between the protonated MMT molecules and the charged metal surface may also contribute to the overall adsorption process in an acidic medium. The thermodynamic and interfacial evaluation of MMT adsorption provides an understanding of its superior protection capabilities.

### 3.9 Excess Surface Charge of the Metal and the Inhibition Mechanism

To examine the electrostatic nature of the metal/electrolyte interface and clarify the proposed adsorption sequence, the excess surface charge of the metal was determined. For this purpose, EIS measurements were carried out at various applied potentials, and the obtained *R*_p_ values were plotted as a function of the applied potential (Figure 10). The potential corresponding to the maximum resistance is defined as the potential of zero charge (*E*_pzc_). According to the approach proposed in the literature, the rational corrosion potential (*E*_r_) is expressed by the following equation:^14,81^

7 $$E_{r}=E_{\mathrm{ocp}}-E_{\mathrm{pzc}}$$

**Figure 10:**
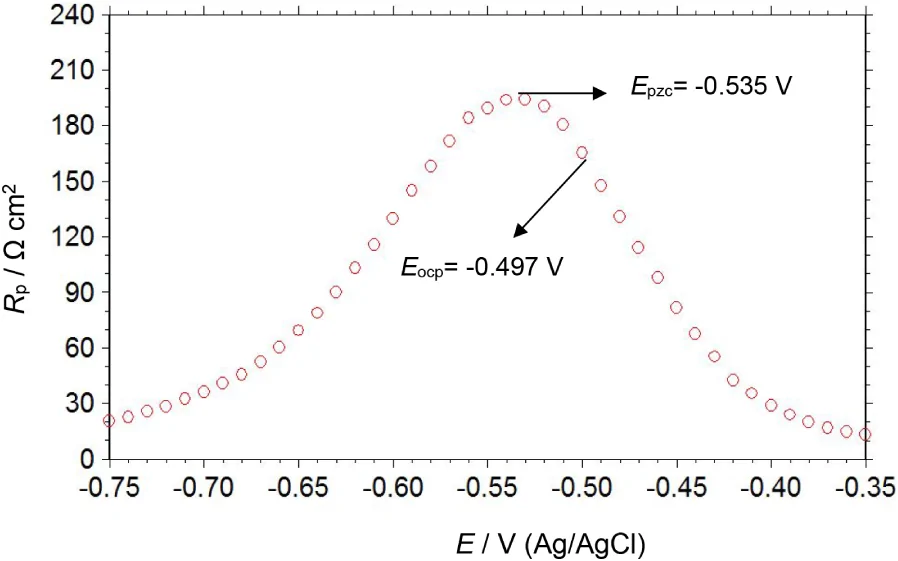
The change of *R*_p_ versus applied potential obtained in the 1 M HCl solution containing 10 mM MMT.

The *R*_p_–*E* plot obtained for the MS in the 1 M HCl solution containing 10 mM MMT is presented in Figure 10. From this curve, the *E*_pzc_ value was determined to be −0.535 V, while the *E*_ocp_ of the electrode under these conditions was found to be −0.497 V. Using these values, *E*_r_ was calculated as +0.038 V. The positive value of *E*_r_ indicates that the surface of the MS in this medium carries an excess positive charge, which is critical for the adsorption sequence.

In HCl solution, the mechanism of the anodic iron dissolution reaction is presented below:^82–84^

8 $${\mathrm{Fe}+\mathrm{Cl}}^{-}↔{({\mathrm{FeCl}}^{-})}_{\mathrm{ads}}$$

9 $${({\mathrm{FeCl}}^{-})}_{\mathrm{ads}}↔{(\mathrm{FeCl})}_{\mathrm{ads}}+e^{-}$$

10 $${(\mathrm{FeCl})}_{\mathrm{ads}}↔\mathrm{FeC}l^{+}+e^{-}$$

11 $${\mathrm{FeCl}}^{+}↔{\mathrm{Fe}}^{2+}{+\mathrm{Cl}}^{-}$$

In acidic media, heterocyclic inhibitor molecules are typically present in their protonated forms in equilibrium with their neutral counterparts, which influences their interaction with the charged metal surface:

12 $${\mathrm{MMT}+H}^{+}↔{\mathrm{MMTH}}^{+}$$

Although the intrinsic surface charge is positive (*E*_r_ > 0), the specific adsorption of Cl^–^ ions can locally modify the interfacial charge distribution as presented in eq 8. Consequently, the protonated MMT molecules (MMTH^+^) interact electrostatically with the MS surface via these preadsorbed Cl^–^ ions, which act as synergistic interconnecting bridges.^85^

13 $${({\mathrm{FeCl}}^{-})}_{\mathrm{ads}}{+\mathrm{MMTH}}^{+}↔{({\mathrm{FeCl}}^{-})}_{\mathrm{ads}}{\mathrm{MMTH}}^{+}$$

The cathodic mechanism of the same process is given below:^82–84^

14 $${\mathrm{Fe}+H}^{+}↔{({\mathrm{FeH}}^{+})}_{\mathrm{ads}}$$

15 $${({\mathrm{FeH}}^{+})}_{\mathrm{ads}}+e^{-}\to {(\mathrm{FeH})}_{\mathrm{ads}}$$

16 $${(\mathrm{FeH})}_{\mathrm{ads}}+H^{+}+e^{-}\to {\mathrm{Fe}+H}_{2}$$

As discussed previously, MMT also acts on cathodic reaction. Therefore, the protonated MMTH^+^ molecules are also adsorbed on cathodic sites in competition with H^+^ ions and mitigate the rate of this reaction.

Following this initial electrostatic attraction, the neutral MMT molecules, which contain electronegative heteroatoms such as N and S, along with an aromatic system rich in π–electrons, serve as potent electron donors.^86^ Therefore, MMT molecules can also subsequently interact directly with the metal surface through coordinate covalent bonds, sharing their lone pairs with the vacant d-orbitals of iron.^60,85–87^ The previously evaluated thermodynamic parameters also align with this multistep proposed mechanism, confirming that the contribution from physical interactions initiates the barrier formation before chemisorption firmly locks the protective film in place.

### 3.10 Theoretical Studies

DFT calculations were performed to reveal the correlations between the structural characteristics of the inhibitor molecule at the atomic level and the experimentally observed inhibition efficiency. According to Frontier molecular orbital theory, the corrosion prevention mechanism is primarily based on donor–acceptor interactions between the inhibitor molecule and the metal surface.^9,60,88^

The energy gap (Δ*E*), electronegativity (χ), and the fraction of electrons transferred (Δ*N*) were calculated using the following equations:^89,90^

17 $$\Delta E=E_{\mathrm{LUMO}}-E_{\mathrm{HOMO}}$$

18 $$\chi =\frac{1}{2}\left(I+A\right)$$

19 $$\eta =\frac{1}{2}\left(I-A\right)$$

20 $$A=-E_{\mathrm{LUMO}}$$

21 $$I=-E_{\mathrm{HOMO}}$$

22 $$\Delta N=\frac{\chi_{\mathrm{Fe}}-\chi_{\mathrm{inh}}}{2\left(\eta_{\mathrm{Fe}}+\eta_{\mathrm{inh}}\right)}$$

where χ_inh_ and χ_Fe_ are the absolute electronegativities of the inhibitor molecules and Fe, *η*_inh_ and *η*_Fe_ are the inhibitor molecule’s global hardness and iron’s global hardness,^91^ and *A* and *I* are the electron affinity and ionization potential, respectively.

Figure 11 shows the optimized 3D geometry of the MMT molecule along with the HOMO and LUMO energy distributions. The relevant quantum chemical parameters are given in Table 4. The data obtained indicate that the MMT molecule interacts with the metal surface predominantly through the active centers discussed earlier. The energy gap (Δ*E*) between the HOMO and LUMO levels is closely related to the molecular polarizability and adsorption ability of the inhibitor molecules on the metal surface. The relatively low Δ*E* value (6.472 eV) of the MMT molecule suggests high molecular reactivity and a minimal energy barrier for electronic transitions, which results in a stronger tendency for interfacial adsorption.^50^

**Figure 11:**
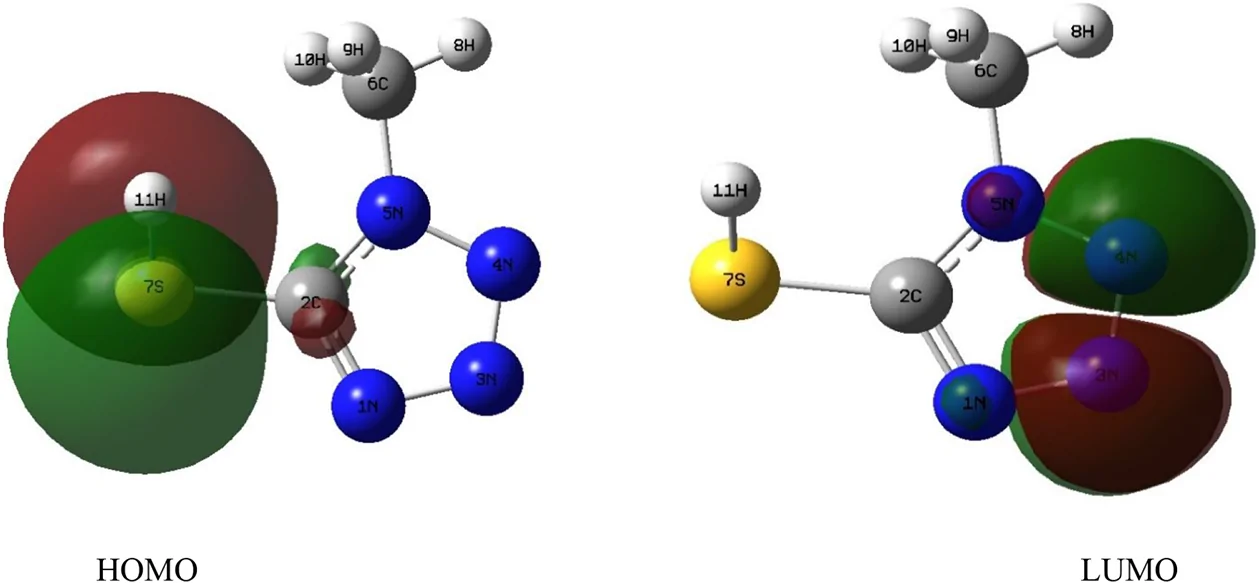
Optimized molecular structure and HOMO and LUMO distributions of MMT.

**Table 4:** Quantum Chemical Parameters Calculated for MMT Using the DFT/B3LYP/6-311++G (d,p) Method

Molecule	*E*_HOMO_/eV	*E*_LUMO_/eV	Δ*E*/eV	χ	Δ*N*	μ/Debye
MMT	–8.110	–1.638	6.472	4.874	0.325	6.291

The value of *E*_HOMO_ (−8.110 eV) for the MMT compound represents a strong tendency of this molecule to donate lone electron pairs from the N and S heteroatoms to the vacant d-orbitals of iron to form coordinate covalent bonds.^40,87,91,92^ Similarly, the low *E*_LUMO_ level (−1.638 eV) of the inhibitor molecule facilitates electron transfer (back-donation) from the filled orbitals of the metal to the empty antibonding orbitals of the inhibitor, synergistically strengthening the overall chemisorption process,^87,90–93^ The calculated energy gap (Δ*E* = 6.472 eV) confirms that the molecule can maintain its chemical stability in a corrosive environment and form a durable protective layer on the metal surface.^94^ This value also supports better reactivity and adsorption tendency of MMT.^91,92^ The calculated absolute electronegativity (χ = 4.874 eV) of the compound is very close to the theoretical work function of the Fe(110) surface (Φ_Fe_ = 4.82 eV), supporting electronic compatibility at the interface. Simultaneously, the positive Δ*N*, fraction of electrons transferred (0.325) value, suggests within the adopted approximation that net electron flow occurs from the inhibitor molecule to the metal surface.^9,61,91,95^

Moreover, the large dipole moment (μ = 6.291 D) of the inhibitor is in full compliance with the physical principle stating that polar compounds are good adsorbents. Molecules with high dipole moments exhibit higher adsorption capacity, enabling more effective adsorption of the molecule at the metal/solution interface.^76,84^ Noteworthy, this value exceeds substantially the dipole moment of an isolated water molecule (1.855 D) indicating a thermodynamically preferred displacement of adsorbed H_2_O molecules by MMT molecules.^67,76^ Overall, the theoretical findings are in good agreement with the experimental observations.

## 4 Conclusions

The inhibitory effect of the heterocyclic MMT compound on the corrosion of MS in the 1 M HCl solution was comprehensively investigated. Electrochemical studies indicated that MMT acts as an efficient corrosion inhibitor, exhibiting concentration-dependent performance with very high protection efficiencies of 94.8% (PDP), 93.0% (EIS), and 92.3% (LPR) at the optimum 10 mM concentration. Surface characterization studies (SEM and EDX) indicated a uniformly distributed, dense, and adherent organic film over the MS surface, which supported the high inhibition efficiency. At lower inhibitor concentrations, MMT acts as a mixed-type inhibitor with dominance of cathodic action. However, at the optimized 10 mM concentration, MMT acts as a mixed-type corrosion inhibitor. After prolonged exposure time, the inhibitor shows a more pronounced effect on the cathodic reaction at the same concentration. The presence of MMT in the HCl solution does not alter the mechanism of the cathodic reaction. In contrast, the inhibitor actively modifies the pathway of the anodic dissolution reaction. The adsorption of MMT molecules on the metal surface obeys the Langmuir isotherm model. The relatively large negative value of Δ*G* ^0^_ads_ (−33.2 kJ/mol) reveals that the adsorption process proceeds via a comprehensive mechanism involving both the physical and chemical interactions. The protective effectiveness of MMT remained greater than 95% even after 120 h of immersion. CA and CV studies indicated excellent electrochemical stability of the MMT surface film under both anodic and cathodic potentials. PZC results and surface characterization studies support a proposed mechanism in which the protonated MMT ions first reach the positively charged metal surface through the preadsorbed chloride bridges and form a highly stable and compact protective layer. DFT computations corroborate the experimental observations at the atomic level. The low energy gap (Δ*E* = 6.472 eV) and the favorable HOMO–LUMO energy distributions theoretically support the high chemical reactivity and the superior electron donor–acceptor capabilities of the MMT molecules.

In conclusion, distinguishing itself from conventional short-term evaluations, this study demonstrates that the novelty of MMT has been evaluated as an effective corrosion inhibitor, offering superior short- and long-term performance in the protection of MS against corrosion in industrial systems operating in HCl environments. Although the remarkable stability and interfacial durability as well as the high protection performance have made MMT a promising material for the prevention of MS corrosion in the 1 M HCl solution, its real large-scale industrial application requires further systematic investigations on its detailed biodegradability tests and overall economic feasibility. Another limitation of the present study is that, although the experimental results demonstrate the promising corrosion inhibition performance of MMT for MS in the 1 M HCl solution under laboratory conditions, longer-term exposure tests in real-world industrial acid cleaning and descaling systems are required before its practical applicability in industrial systems can be reliably established.

## Data Availability

The data underlying this study are available from the corresponding author upon reasonable request.
